# Effectiveness of Al-Assisted Patient Health Education Using Voice Cloning and ChatGPT: Prospective Randomized Controlled Trial

**DOI:** 10.2196/81387

**Published:** 2026-03-19

**Authors:** Yan Sun, Shangqing Xu, Hongying Jin, Xiaoyan Han, Kangqi Jin, Yimei Zhang, Xiaoli Ma, Huaping Wei, Minjie Ma

**Affiliations:** 1 Department of Thoracic Surgery, The First Hospital of Lanzhou University, Donggang West Road 1#, Lanzhou, 730000, China; 2 School of Nursing, Lanzhou University, Lanzhou, China; 3 Outpatient Department, The First Hospital of Lanzhou University, Lanzhou, China; 4 Gansu International Science and Technology Cooperation Base for Development and Application of Thoracic Surgery Key Technologies, The First Clinical Medical College of Lanzhou University, Department of Thoracic Surgery, The First Hospital of Lanzhou University, Donggang West Road 1#, Lanzhou, 730000, China, 86 13639325950

**Keywords:** artificial intelligence, voice cloning, medical education, ChatGPT, education effect evaluation, randomized controlled trial

## Abstract

**Background:**

Traditional patient education often lacks personalization and engagement, potentially limiting knowledge acquisition and treatment adherence. Advances in artificial intelligence (AI), including voice cloning technology and large language models (eg, ChatGPT), offer new opportunities to deliver personalized, scalable, interactive health education. However, evidence regarding the comparative effectiveness of different AI-based voice cloning strategies and reliability of automated AI evaluation tools remains limited.

**Objective:**

This study aims to evaluate the effectiveness of AI-assisted patient education integrating voice cloning and ChatGPT, compare physician voice cloning with patient self-voice cloning, and assess the reliability of ChatGPT as an automated evaluation tool for education outcomes.

**Methods:**

In this prospective, 3-arm, parallel-group randomized controlled trial, 180 hospitalized patients requiring standardized health education were recruited from a tertiary hospital. Inclusion criteria were age ≥18 years, clear diagnosis requiring health education, clear consciousness, and voluntary participation with informed consent. Exclusion criteria were severe hearing impairment, severe cognitive impairment, expected hospitalization <3 days, or prior participation in similar studies. Using a computer-generated random sequence, participants were randomly assigned (1:1:1) to receive traditional education (control), AI-assisted education using physician voice cloning, or AI-assisted education using patient self-voice cloning, each with identical educational content of equal duration. The primary outcome was education content compliance, evaluated using ChatGPT-4 with validated prompts and verified by expert review. Secondary outcomes included knowledge retention, education satisfaction, treatment adherence, quality of life, and psychological status. Outcome assessors and data analysts, but not participants, were blinded to group allocation.

**Results:**

Of 180 randomized participants, 174 (96.7%) completed the trial. Both AI-assisted groups had significantly higher mean education content compliance scores immediately posteducation than the control group (physician voice: 86.7, SD 7.3; self-voice: 92.5, SD 6.8; control: 73.2, SD 8.5; *P*<.001). The patient self-voice group showed superior predischarge knowledge retention, higher education satisfaction, and greater treatment adherence than the other groups (all *P*≤.02). At the 1-month follow-up, the self-voice group maintained improved adherence (Cohen *d*=0.74) and had significantly lower anxiety and depression scores (all *P*≤.02) and improved SF-36 quality-of-life domains. ChatGPT-based evaluations demonstrated high reliability (weighted κ=0.87, 95% CI 0.82‐0.91)^.^

**Conclusions:**

The innovative patient education model integrating AI voice cloning and ChatGPT is distinct from previous studies primarily relying on standard text-to-speech or professionally recorded content. Using patients’ own cloned voices for health education delivery leveraged the self-reference effect to enhance learning outcomes. Compared with research using clinician-narrated content, this study highlights that self-voice education produces superior outcomes across multiple domains including compliance, satisfaction, and psychological well-being. These findings establish a theoretical and practical framework for personalized AI-driven patient education. In real-world clinical settings, this approach offers a scalable, cost-effective solution to enhance patient engagement, particularly valuable in resource-limited environments where individualized education is challenging to deliver.

## Introduction

Patient education is a fundamental component of health care, aiming to enhance individuals’ understanding of their medical conditions, promote self-management, improve treatment adherence, and ultimately optimize health outcomes and prognosis [1-3]. Despite the clinical significance of patient education, traditional methods of patient education—primarily verbal explanations by health care providers and standardized written materials—often fall short in addressing the diverse needs of patients [4,5]. These approaches are constrained by limited time, lack of interactivity, and insufficient personalization, frequently resulting in suboptimal comprehension, poor engagement, and reduced adherence to treatment recommendations [6].

Prior research has shown that personalized, interactive education enhances patient understanding and engagement [7-11]. Recent advances in artificial intelligence (AI), particularly in voice cloning and large language models (LLMs), present novel opportunities to transform conventional health education into a more personalized, scalable, and interactive process [12]. Voice cloning technology uses deep learning algorithms to synthesize natural-sounding speech from limited voice samples, enabling the delivery of educational content in familiar voices—such as those of physicians or even the patients themselves [13,14]. Prior studies suggest that familiar voices can increase emotional resonance [15-17], build trust, enhance patient engagement, improve memory retention of medical information [15,17], and increase accessibility to digital health tools. Recent scoping reviews have identified multiple applications of LLMs in patient education, including generating educational materials, interpreting medical information, and optimizing doctor-patient interaction [18]. Systematic reviews have further demonstrated that LLMs can enhance health care communication and support personalized patient engagement [19].

Simultaneously, LLMs like ChatGPT, which is based on advanced LLMs, have demonstrated strong capabilities for natural language understanding, dialog generation, and semantic evaluation [20] and have shown potential for supporting patient communication and education [18,19,21-23]. These tools can interact with patients in real time, deliver conversational-style education, and assess understanding through dynamic, automated evaluation processes [24-26]. Such integration may address key challenges in health education, including the need for tailored content delivery and standardized assessment without overburdening clinical staff. These models are capable of few-shot learning, enabling rapid adaptation to novel prompts [27].

Despite this emerging literature, significant gaps remain. First, few empirical studies have rigorously examined the comparative effectiveness of different voice sources—specifically physician-cloned voice versus patient-cloned voice—in AI-assisted patient education. Second, although LLM-based tools like ChatGPT show promise for health communication, their role as standardized evaluators of educational outcomes has not been comprehensively validated in clinical randomized trials. Traditional outcome assessments often rely on human raters, which introduces inter-rater variability and resource burdens.

To address these gaps, this study was a prospective randomized controlled trial (RCT) to investigate the effectiveness of an AI-assisted patient education system integrating voice cloning and ChatGPT. The study compared educational outcomes between physician voice cloning and patient self-voice cloning and explored the feasibility and accuracy of using ChatGPT as a standardized, automated evaluation tool. By doing so, this work aimed to contribute novel insights into how AI technologies can enhance the personalization, efficiency, and effectiveness of inpatient education and to extend the evidence base for AI-enabled patient engagement strategies. We hypothesized that (1) AI-assisted voice cloning education would significantly improve patient education outcomes compared with traditional education, (2) patient self-voice cloning would yield superior educational effectiveness compared with physician voice cloning due to the self-reference effect, and (3) ChatGPT could serve as a reliable automated evaluation tool with high agreement with expert assessment.

## Methods

### Overall Study

This study used an RCT design to assess the impact of AI-assisted patient health education using voice cloning technology and ChatGPT on patient education outcomes. Participants were randomly assigned to 1 of 3 groups: The 3 groups received different educational methods but the same educational content and duration of education. Following the educational intervention, the effectiveness of the education was comprehensively evaluated at fixed time points using ChatGPT. To ensure the validity and accuracy of the evaluation tool, a pre-test of the ChatGPT evaluation tool was conducted prior to the formal data collection. This study not only explored the potential of AI voice cloning technology in personalized patient education but also used ChatGPT as an assessment tool to further validate its reliability and practicality in evaluating health education interventions. Ultimately, the study aimed to provide theoretical insights and practical recommendations for improving medical health education models, enhancing patient education outcomes, and promoting treatment adherence and quality of life.

### Study Timeline

An independent tool validation study was conducted from January 2024 to June 2024 to verify the ChatGPT-based compliance scoring tool (no participant enrollment). Administrative preparation, staff training, and software and hardware debugging were completed in December 2024, and this period did not involve participant enrollment. Trial registration (ChiCTR2500101882) was initiated on January 15, 2025, and finalized on April 30, 2025, before enrollment began in May 2025. Participant enrollment and intervention delivery were conducted from May 2025 to June 2025, with 1-month follow-up assessments completed by July 2025.

### Reporting Guidelines

The trial was conducted and reported in accordance with the CONSORT (Consolidated Standards of Reporting Trials) 2025 statement and the CONSORT-EHEALTH checklist for digital and AI-based health interventions (Checklists1  2) [28,29].

### Study Design

This single-center, 3-arm, parallel-group, superiority RCT (allocation ratio 1:1:1) was conducted at The First Hospital of Lanzhou University. System development, intervention standardization, and staff training were completed in December 2024 and January 2025. Participant recruitment and data collection were conducted from May 2025 to July 2025.

### Randomization

#### Sequence Generation

The random allocation sequence was generated using a computer-generated random number table.

#### Allocation Concealment

To ensure concealment of group assignments, the allocation sequence was placed in sealed, opaque envelopes managed by independent researchers who were not involved in the implementation of the interventions. This procedure minimized the risk of selection bias.

#### Implementation

Independent researchers generated the allocation sequence and managed the envelopes. Eligible participants were assigned to study arms according to the contents of the envelopes.

#### Blinding

Due to the nature of the interventions, neither participants nor the medical staff implementing the interventions could be blinded. However, outcome assessors were blinded to group assignments to minimize assessment bias.

### Ethical Considerations

The clinical trial protocol was reviewed and approved by the Ethics Committee of The First Hospital of Lanzhou University (approval number: LDYYLL-2025‐805) and conducted in accordance with the principles of the Declaration of Helsinki.

All participants received detailed verbal and written information regarding the study purpose, procedures, potential risks, and expected benefits and provided written informed consent prior to enrollment. The consent process also covered permission for the use and publication of anonymized data. Participants were informed that their involvement was voluntary and that they could withdraw from the study at any time without affecting their standard medical care.

All collected data were deidentified before analysis and stored in encrypted, password-protected databases accessible only to the research team. Personal identifiers were removed to ensure participant confidentiality and data security.

The use of voice cloning technology was explicitly covered in the consent form. Participants and physicians provided separate written authorization for the use of their voice recordings solely for research purposes. All voice samples and generated models were permanently deleted after study completion.

Participants did not receive monetary compensation but were offered free access to the AI-based health education program and follow-up consultations as part of their clinical care. No identifiable photographs nor audiovisual materials were used in this publication.

### Participants

The study participants were patients hospitalized in a tertiary hospital from May 2025 to July 2025 who needed to receive medical education.

### Inclusion and Exclusion Criteria

The inclusion criteria were (1) age ≥18 years of any gender; (2) clear diagnosis, patients who needed to receive medical education; (3) clear consciousness, able to understand and cooperate with the study; and (4) voluntary participation in this study and signed informed consent. The exclusion criteria were (1) severe hearing impairment, unable to normally receive voice education; (2) severe cognitive impairment, unable to understand education content; (3) expected hospitalization time <3 days; and (4) previously participated in similar studies.

### Sample Size

Based on the pre-experimental results, with the education content compliance rate as the main observation indicator, *α* set at 0.05 (2-sided), *β* set at 0.10, a test power of 90%, and an expected effect size of 0.3 among the 3 groups, we calculated that each group needed a sample size of 54 participants using G*Power 3.1 software. Considering a possible dropout rate of approximately 10%, we finally determined that we would enroll 60 participants in each group, for a total of 180 participants. Prior to participation, all patients were informed that the educational intervention would be delivered using AI-generated voice recordings. Written informed consent was obtained from all participants.

### Independent Tool Validation Study (January 2024 to June 2024): Validation of ChatGPT-Based Compliance Scoring

This independent tool validation study was conducted separately from the randomized trial and did not involve participant enrollment. To validate the AI-based scoring used for the primary outcome, we conducted a separate pilot validation study (January 2024 through June 2024) among 30 volunteers who were not part of the randomized trial. Phase 1 (verbatim-matching workflow) used iFlytek automatic speech recognition (Mandarin medical context; no manual correction) to transcribe each participant’s recitation; the transcribed text was pasted into the ChatGPT web interface together with the standard education text for rubric-based scoring. Phase 2 (semantic audio-based workflow) re-evaluated the same recordings by uploading the original audio directly to the ChatGPT web interface (WAV/MP3; 16-kHz sampling rate; 60‐120 s per recording); each recording was scored once. Both workflows were benchmarked against consensus ratings from 3 senior nursing experts using the same rubric. Agreement with expert ratings was substantial in Phase 1 (weighted κ=.72) and higher in Phase 2 (weighted κ=.87), with strong internal consistency of the evaluation items (Cronbach *α*=0.89). Therefore, Phase 2 was selected for the main RCT to minimize transcription-related errors and enhance reproducibility. Detailed prompts, rubric, and example inputs and outputs are provided in Multimedia Appendix 1.

### Intervention Measures

#### Traditional Education Group (Control Group)

The traditional education group received the hospital’s current standardized patient education, including verbal education by medical staff and standardized written education materials. Verbal education was conducted by trained medical staff according to a unified education outline, including disease knowledge, treatment plans, precautions, and lifestyle guidance. Written education materials were standardized education manuals compiled by the hospital that patients could take back to the ward or home to read. The duration of the education was approximately 20 minutes to 30 minutes.

#### Attending Physician Voice Cloning Education Group (Intervention Group 1)

The physician voice cloning education group (intervention group 1) received patient education through cloning of the attending physician’s voice. First, the patient’s attending physician’s voice sample (approximately 5 min) was obtained, and Resemble.ai voice cloning technology was used to generate the doctor’s voice model. Medical professionals then formulated personalized education content based on standard education content and the patient’s specific situation, synthesized the voice using doctor voice cloning, and produced personalized voice education materials. Patients listened to the voice-cloned education content through headphones or speakers under the guidance of research assistants and received printed personalized education materials. The education content covered the same topics as the control group but was personalized according to patient characteristics, such as using language expressions suitable for the patient’s education level, lifestyle suggestions targeted at the patient’s occupational characteristics, and emphasizing relevant content based on the patient’s concerns. The duration of the education was approximately 20 minutes to 30 minutes.

#### Patient’s Own Voice Cloning Education Group (Intervention Group 2)

The patient voice cloning education group received patient education through cloning of the patient’s own voice. First, the patient’s voice sample (approximately 5 min) was obtained, and Resemble.ai voice cloning technology was used to generate the patient’s voice model. Similar to intervention group 1, medical professionals then formulated personalized education content, synthesized the voice through patient voice cloning, and produced personalized voice education materials. Patients listened to the voice-cloned education content through mobile phone headphones or speakers under the guidance of research assistants and received printed personalized education materials. The education content was the same as in intervention group 1, with the only difference being the use of the patient’s own voice for education. The duration of the education was approximately 20 minutes to 30 minutes.

#### Educational Content and Assessment of Education Effect

All participants received the same educational content and could ask questions after receiving education, with medical staff answering inquiries. All 3 groups of patients had their education effect assessed before discharge by using ChatGPT to compare the education content recited by patients with the original education content to calculate the content compliance rate, and follow-up assessments were conducted 1 month after discharge.

The educational intervention included AI-generated voice recordings delivered using cloning of either the attending physician’s voice or the patient’s own voice. ChatGPT was used to assess patient knowledge, satisfaction, and adherence using standardized prompts. Patient outcomes were also measured using structured questionnaires. The questionnaires are provided in Multimedia Appendices 2-4, and the voice cloning patient education prompts, ChatGPT evaluation prompts, and AI-generated educational content are provided in Multimedia Appendix 5. Data were collected at baseline and postintervention, and all responses were recorded in a secure electronic database.

### Evaluation Indicators and Measurement Tools

#### Primary Outcome Measure

The primary outcome, education content compliance rate, was defined as the degree of semantic concordance between the education content recited by patients and the standardized education material using the following equation: compliance rate = (number of key points correctly recited by patients/total number of key points in education content) × 100%. The education content compliance rate was assessed using ChatGPT-4 (model ChatGPT [OpenAI; web interface, model displayed as GPT-4 during the study period]) as an automated evaluation tool to quantify semantic similarity between the patient’s oral recitation and the standard education content, enabling scalable and objective scoring.

#### Secondary Outcome Measures

##### Knowledge Mastery

We used disease-related knowledge questionnaires to assess patients’ understanding and memory of disease knowledge, with a possible total score of 100 points and higher scores indicating better knowledge mastery.

##### Education Satisfaction

We used the education satisfaction scale to assess patients’ satisfaction with the education process and content, including aspects such as education content, method, time, and effect, using a 5-level Likert scale. Higher scores indicate higher satisfaction.

##### Treatment Adherence

We used the treatment adherence scale to assess patients’ treatment adherence within 1 month after discharge, including aspects such as drug treatment, lifestyle adjustment, and follow-up review. Higher scores indicate better adherence.

##### Quality of Life

We used the SF-36 quality of life scale to assess changes in patients’ quality of life 1 month after discharge.

##### Anxiety and Depression

We used the Hospital Anxiety and Depression Scale to assess changes in patients’ anxiety and depression before and after the education.

### Evaluation Time Points

#### Baseline Assessment

We collected patients’ basic information and baseline data, including demographic characteristics, disease-related knowledge, anxiety, and depression, before the intervention.

#### Immediate Posteducation Assessment

The education content compliance rate, knowledge mastery, and education satisfaction were assessed immediately after the education was completed.

#### Predischarge Assessment

Education content compliance rate and knowledge mastery were re-assessed 1 day before patient discharge.

#### 1-Month Postdischarge Follow-Up Assessment

Through telephone or outpatient follow-up, we assessed patients’ treatment adherence, quality of life, disease-related knowledge retention, anxiety, and depression.

### Data Collection and Management

Research assistants who had received standardized training were responsible for data collection, using uniform data collection forms to record patient information and evaluation results. An electronic database was established, with all data independently entered by 2 personnel and cross-checked to ensure accuracy. To protect patient privacy, all data were stored in coded form, and regular data quality checks were conducted to ensure completeness and reliability. Missing data were promptly identified and imputed.

All voice recordings collected in this study were considered sensitive personal data. They were stored on secure, password-protected servers at The First Hospital of Lanzhou University, accessible only to authorized research personnel. For analysis, the recordings were anonymized. Following the completion of the study, all original recordings will be permanently deleted in accordance with institutional data retention and privacy policies.

### Statistical Analysis

Statistical analyses were performed using SPSS version 26.0 (IBM Corp). All analyses followed the intention-to-treat (ITT) principle. Missing outcome data (6/182, 3.3%) were evaluated using the missing completely at random test from Little, which indicated that missingness was completely at random (*χ*²_12_=14.5, *P*=.27). Consequently, multiple imputation by chained equations was used to generate 5 imputed datasets (m=5), and pooled estimates were obtained using the rules by Rubin. Sensitivity analyses using per-protocol (complete-case) data yielded consistent findings (absolute difference in mean compliance<1%). Continuous variables are presented as mean (SD), and categorical variables are presented as n (%). Between-group comparisons were conducted using 1-way ANOVA with post hoc tests (least significant difference or Bonferroni, as appropriate). Categorical variables were compared using χ² tests or Fisher exact tests. All tests were 2-sided with a significance level of *P*<.05.

### Evaluation Indicators

The primary outcome of this study was the education content compliance rate, which reflected how well patients understood and followed the health education provided. Secondary outcomes included knowledge retention, patient satisfaction, and treatment adherence.

Education level and knowledge retention were measured using a structured questionnaire developed by the research team based on the hospital’s standardized education materials.

The questionnaire consisted of multiple-choice and short-answer questions assessing patients’ understanding and recall of the key educational points immediately after education and before discharge.

Each correct response was scored as 1 point, with higher total scores indicating better knowledge retention.

The disease-related knowledge questionnaire was developed based on the hospital’s standardized education materials and underwent content review by 3 senior nursing experts.

## Results

### Participant Flow

**Figure 1.**
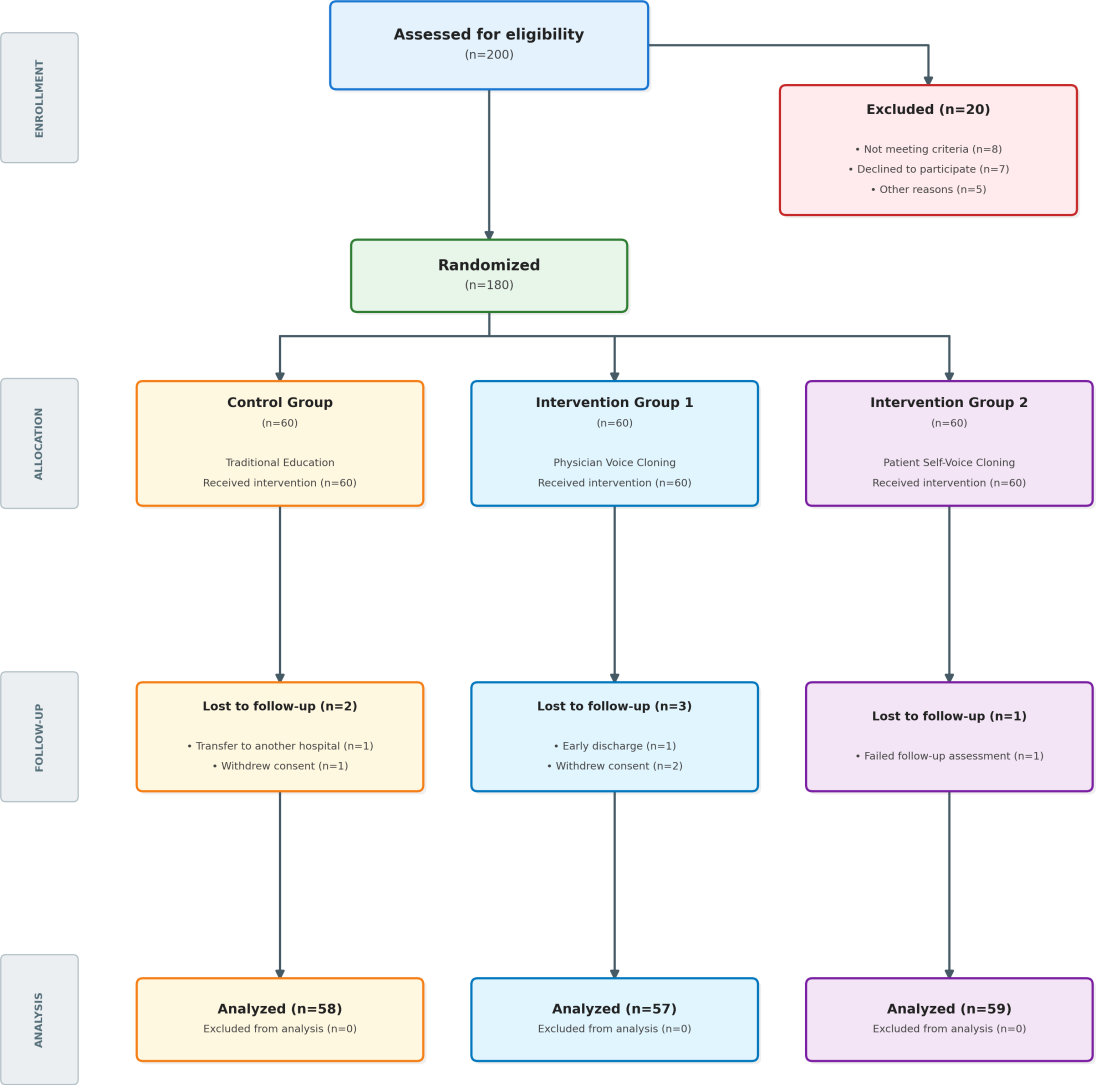
CONSORT (Consolidated Standards of Reporting Trials) flow diagram.

Figure 1 illustrates the participant flow. ITT analysis was performed for all participants (n=180) using multiple imputation by chained equations for missing data. The analysis section in the figure shows the per-protocol analyses (n=174).

### Comparison of General Information

A total of 180 patients were recruited to participate in this study. During the follow-up period, 6 participants were lost to follow-up due to transfer to another hospital (n=1) and withdrawal of consent (n=1) in the control group; early discharge (n=1) and withdrawal of consent (n=2) in intervention group 1; and failure to complete follow-up assessments (n=1) in intervention group 2. Therefore, 174 patients completed the study (58 in the control group, 57 in intervention group 1, 59 in intervention group 2), for a total dropout rate of 3.3% (6/180). After multiple imputation, the analysis results remained consistent with those of the complete-case dataset, indicating the robustness of the findings. Comparison of general information such as age, gender, education level, occupation, and disease type among the 3 groups of patients showed no statistically significant differences (*P*>.05), indicating comparability (Table 1).

**Table 1.** Comparison of baseline characteristics among 3 groups of hospitalized patients with lung cancer in a randomized controlled trial of artificial intelligence (AI)–assisted voice cloning education at The First Hospital of Lanzhou University (May 2025 through July 2025).

Item	Control group (n=58)	Intervention group 1 (n=57)	Intervention group 2 (n=59)	Statistical test result (*df*)	*P* value
Age (years), mean (SD)	52.6 (14.8)	53.2 (15.1)	51.9 (14.5)	0.127^a^ (2, 171)	.88
Gender, n (%)				0.215^b^ (2)	.90
Male	31 (53)	32 (56)	34 (58)		
Female	27 (47)	25 (44)	25 (42)		
Education level, n (%)				0.986^b^ (6)	.91
Primary school and less	12 (21)	11 (19)	10 (17)		
Junior high school	18 (31)	17 (30)	19 (32)		
High school/technical secondary school	16 (28)	18 (32)	17 (29)		
College and more	12 (21)	11 (19)	13 (22)		
Marital status, n (%)				0.352^b^ (2)	.84
Married	49 (85)	47 (83)	51 (86)		
Unmarried/divorced/widowed	9 (16)	10 (18)	8 (14)		
Disease type, n (%)				0.763^b^ (6)	.94
Cardiovascular disease	21 (36)	20 (35)	22 (37)		
Respiratory system disease	15 (26)	16 (28)	17 (29)		
Digestive system disease	13 (22)	12 (21)	11 (19)		
Others	9 (16)	9 (16)	9 (15)		
Previous hospitalization history, n (%)				0.124^b^ (2)	.94
Yes	32 (55)	33 (58)	33 (56)		
No	26 (45)	24 (42)	26 (44)		
Baseline HADS^c^ score, mean (SD)					
Anxiety	7.8 (3.2)	7.6 (3.1)	7.7 (3.3)	0.068^a^ (2, 171)	.93
Depression	6.9 (2.8)	7.1 (2.9)	6.8 (2.7)	0.185^a^ (2, 171)	.83

a *F* test.

b Chi-square test.

c HADS: Hospital Anxiety and Depression Scale.

### Comparison of Education Content Compliance Rate

Immediately after education, the mean education compliance score in the control group was 73.2 (SD 8.5; 95% CI 71.0‐75.4), compared with mean scores of 86.7 (SD 7.3; 95% CI 84.8‐88.6) in intervention group 1 and 92.5 (SD 6.8; 95% CI 90.8‐94.2) in intervention group 2. The 1-way ANOVA showed a statistically significant difference among the 3 groups (*F*_2,171_=103.427, *P*<.001). Before discharge, the mean score in the control group was 68.5 (SD 9.1; 95% CI 66.2‐70.8), while the mean scores in intervention group 1 and intervention group 2 were 82.3 (SD 8.1; 95% CI 80.2‐84.4) and 88.6 (SD 7.4; 95% CI 86.7‐90.5), respectively. The between-group difference remained statistically significant (*F*_2,171_=95.682, *P*<.001; Table 2).

**Table 2.** Comparison of education content compliance rate among 3 groups of hospitalized patients with lung cancer in a randomized controlled trial of artificial intelligence (AI)–assisted voice cloning education at The First Hospital of Lanzhou University (May 2025 through July 2025), with differences analyzed using 1-way ANOVA.

Time point	Control group (n=58), mean (SD; 95% CI)	Intervention group 1 (n=57), mean (SD; 95% CI)	Intervention group 2 (n=59), mean (SD; 95% CI)	*F* test (*df*)	*P* value
Immediately after education	73.2 (8.5; 71.0‐75.4)	86.7 (7.3; 84.8‐88.6)	92.5 (6.8; 90.8‐94.2)	103.427 (2, 171)	<.001
Before discharge	68.5 (9.1; 66.2‐70.8)	82.3 (8.1; 80.2‐84.4)	88.6 (7.4; 86.7‐90.5)	95.682 (2, 171)	<.001

The corresponding ITT results based on the multiply imputed datasets are provided in Supplementary Table S2.

### Comparison of Knowledge Mastery

Immediately after education, the mean knowledge mastery score in the control group was 76.3 (SD 9.2; 95% CI 73.9‐78.7), which was significantly lower than the mean scores in intervention group 1 (88.5, SD 8.4; 95% CI 86.3‐90.7) and intervention group 2 (90.2, SD 8.1; 95% CI 88.1‐92.3). A statistically significant difference was observed among the 3 groups (*F*_2,171_=48.362, *P*<.001). Before discharge, the control group had a mean knowledge mastery score of 71.8 (SD 9.8; 95% CI 69.2‐74.4), whereas intervention group 1 and intervention group 2 achieved higher mean scores of 84.7 (SD 8.9; 95% CI 82.3‐87.1) and 87.3 (SD 8.5; 95% CI 85.1‐89.5), respectively. The between-group difference remained statistically significant (*F*_2,171_=46.175, *P*<.001). At 1 month after discharge, the mean knowledge mastery score further declined in the control group (65.2, SD 10.5; 95% CI 62.4‐68.0). In contrast, both intervention groups maintained relatively higher levels of knowledge mastery, with mean scores of 78.6 (SD 9.3; 95% CI 76.1‐81.1) in intervention group 1 and 83.5 (SD 8.9; 95% CI 81.2‐85.8) in intervention group 2. The overall difference among the 3 groups remained statistically significant (*F*_2,171_=54.793, *P*<.001; Table 3).

**Table 3.** Comparison of knowledge mastery scores among 3 groups of hospitalized patients with lung cancer in a randomized controlled trial of artificial intelligence (AI)–assisted voice cloning education at The First Hospital of Lanzhou University (May 2025 through July 2025), with differences among groups analyzed using 1-way ANOVA.

Time point	Control group (n=58), mean (SD; 95% CI)	Intervention group 1 (n=57), mean (SD; 95% CI)	Intervention group 2 (n=59), mean (SD; 95% CI)	*F* test (*df*)	*P* value
Immediately after education	76.3 (9.2; 73.9-78.7)	88.5 (8.4; 86.3-90.7)	90.2 (8.1; 88.1-92.3)	48.362 (2, 171)	<.001
Before discharge	71.8 (9.8; 69.2-74.4)	84.7 (8.9; 82.3-87.1)	87.3 (8.5; 85.1-89.5)	46.175 (2, 171)	<.001
1 month after discharge	65.2 (10.5; 62.4-68.0)	78.6 (9.3; 76.1-81.1)	83.5 (8.9; 81.2-85.8)	54.793 (2, 171)	<.001

### Comparison of Education Satisfaction

Significant differences were observed among the 3 groups across all dimensions of education satisfaction, including education content, education method, education time, education effect, and overall satisfaction (all *P*<.001). Across all items, the 95% CIs of the 2 intervention groups were consistently higher than those of the control group, with minimal overlap between groups. This indicates that the observed differences in education satisfaction were not only statistically significant but also stable and precise, reflecting a reliable improvement associated with the intervention (Table 4).

**Table 4.** Comparison of education satisfaction scores among 3 groups of hospitalized patients with lung cancer in a randomized controlled trial of artificial intelligence (AI)–assisted voice cloning education at The First Hospital of Lanzhou University (May 2025 through July 2025), with differences among groups analyzed using 1-way ANOVA.

Item	Control group (n=58), mean (SD; 95% CI)	Intervention group 1 (n=57), mean (SD; 95% CI)	Intervention group 2 (n=59), mean (SD; 95% CI)	*F* test (*df*)	*P* value
Education content	3.8 (0.7; 3.6‐4.0)	4.3 (0.6; 4.1‐4.5)	4.5 (0.5; 4.4‐4.6)	21.364 (2, 171)	<.001
Education method	3.6 (0.8; 3.4‐3.8)	4.5 (0.6; 4.3‐4.7)	4.7 (0.4; 4.6‐4.8)	52.781 (2, 171)	<.001
Education time	3.9 (0.6; 3.7‐4.1)	4.2 (0.5; 4.1‐4.3)	4.3 (0.5; 4.2‐4.4)	9.526 (2, 171)	<.001
Education effect	3.7 (0.7; 3.5‐3.9)	4.4 (0.5; 4.3‐4.5)	4.6 (0.4; 4.5‐4.7)	43.175 (2, 171)	<.001
Overall satisfaction	3.8 (0.6; 3.6‐4.0)	4.4 (0.5; 4.3‐4.5)	4.6 (0.4; 4.5‐4.7)	45.293 (2, 171)	<.001

### Comparison of Treatment Adherence

Among the 3 groups, significant differences in treatment adherence were observed across all dimensions, including drug treatment adherence, lifestyle adjustment, follow-up review, and total adherence score (all *P*<.001). For drug treatment adherence, the control group scored a mean of 7.6 (SD 1.5; 95% CI 7.2‐8.0), which was lower than the mean scores for intervention group 1 (8.5, SD 1.2; 95% CI 8.2‐8.8) and intervention group 2 (9.1, SD 0.9; 95% CI 8.9‐9.3), with a statistically significant overall difference (*F*_2,171_=25.364, *P*<.001). Similar patterns were observed for lifestyle adjustment and follow-up review adherence. The 95% CIs of both intervention groups were consistently higher than those of the control group, with limited overlap, indicating that the observed improvements in treatment adherence were stable and precise. Regarding the total adherence score, the control group achieved a mean of 21.6 (SD 4.2; 95% CI 20.5‐22.7), whereas intervention group 1 and intervention group 2 achieved higher mean scores of 24.7 (SD 3.5; 95% CI 23.8‐25.6) and 26.6 (SD 2.8; 95% CI 25.9‐27.3), respectively. The between-group difference remained statistically significant (*F*_2,171_=32.593, *P*<.001; Table 5).

**Table 5.** Comparison of treatment adherence scores among 3 groups of hospitalized patients with lung cancer in a randomized controlled trial of artificial intelligence (AI)–assisted voice cloning education at The First Hospital of Lanzhou University (May 2025 through July 2025), with differences among groups analyzed using 1-way ANOVA.

Item	Control group (n=58), mean (SD; 95% CI)	Intervention group 1 (n=57), mean (SD; 95% CI)	Intervention group 2 (n=59), mean (SD; 95% CI)	*F* test (*df*)	*P* value
Drug treatment	7.6 (1.5; 7.2‐8.0)	8.5 (1.2; 8.2‐8.8)	9.1 (0.9; 8.9‐9.3)	25.364 (2, 171)	<.001
Lifestyle adjustment	6.8 (1.7; 6.4‐7.2)	7.9 (1.4; 7.5‐8.3)	8.6 (1.1; 8.3‐8.9)	27.175 (2, 171)	<.001
Follow-up review	7.2 (1.6; 6.8‐7.6)	8.3 (1.3; 8.0‐8.6)	8.9 (1.0; 8.6‐9.2)	26.482 (2, 171)	<.001
Total score	21.6 (4.2; 20.5‐22.7)	24.7 (3.5; 23.8‐25.6)	26.6 (2.8; 25.9‐27.3)	32.593 (2, 171)	<.001

### Comparison of Quality of Life

One month after discharge, significant differences were observed among the 3 groups in all SF-36 dimensions, including physical functioning, role-physical, bodily pain, general health, vitality, social functioning, role-emotional, and mental health (all *P*<.001). For each dimension, the 95% CIs of both intervention groups were consistently higher than those of the control group, indicating that the observed improvements in health-related quality of life were robust and precise.

Notably, intervention group 2 generally showed narrower 95% CIs, suggesting less variability and greater consistency in patient-reported outcomes compared with the control group (Table 6).

**Table 6.** Comparison of SF-36 quality of life scores 1 month after discharge among 3 groups of hospitalized patients with lung cancer in a randomized controlled trial of artificial intelligence (AI)–assisted voice cloning education at The First Hospital of Lanzhou University (May 2025 through July 2025), with differences among groups analyzed using 1-way ANOVA.

Dimension	Control group (n=58), mean (SD; 95% CI)	Intervention group 1 (n=57), mean (SD; 95% CI)	Intervention group 2 (n=59), mean (SD; 95% CI)	*F* test (*df*)	*P* value
Physical functioning	65.3 (12.5; 61.7‐68.9)	72.6 (11.3; 69.5‐75.7)	74.8 (10.9; 71.8‐77.8)	11.364 (2, 171)	<.001
Role-physical	58.6 (14.2; 54.4‐62.8)	67.5 (12.8; 63.8‐71.2)	70.3 (12.1; 66.9‐73.7)	13.175 (2, 171)	<.001
Bodily pain	67.2 (13.1; 63.3‐71.1)	74.8 (11.9; 71.5‐78.1)	76.5 (11.2; 73.3‐79.7)	10.482 (2, 171)	<.001
General health	61.5 (12.8; 57.7‐65.3)	69.7 (11.5; 66.5‐72.9)	72.4 (10.8; 69.3‐75.5)	14.293 (2, 171)	<.001
Vitality	59.8 (13.5; 55.8‐63.8)	68.3 (12.2; 65.1‐71.5)	71.6 (11.5; 68.5‐74.7)	15.364 (2, 171)	<.001
Social functioning	63.7 (12.9; 59.8‐67.6)	71.5 (11.7; 68.4‐74.6)	76.2 (10.8; 73.2‐79.2)	18.175 (2, 171)	<.001
Role-emotional	60.2 (14.1; 56.0‐64.4)	69.8 (12.5; 66.3‐73.3)	75.3 (11.7; 72.1‐78.5)	21.482 (2, 171)	<.001
Mental health	62.5 (13.2; 58.7‐66.3)	70.6 (12.1; 67.3‐73.9)	76.8 (11.3; 73.8‐79.8)	22.293 (2, 171)	<.001

### Comparison of Anxiety and Depression

Before education, there were no significant differences in anxiety nor depression scores among the 3 groups (anxiety: *F*_2,171_=0.068, *P*=.93; depression: *F*_2,171_=0.185, *P*=.83), indicating baseline comparability. One month after discharge, significant differences were observed among the groups for both anxiety and depression scores (*P*<.001). Specifically, for anxiety, the control group scored a mean of 6.5 (SD 2.9; 95% CI 5.8‐7.2), intervention group 1 scored a mean of 5.2 (SD 2.5; 95% CI 4.6‐5.8), and intervention group 2 scored a mean of 4.3 (SD 2.2; 95% CI 3.8‐4.8). For depression, the mean scores were 5.8 (SD 2.6; 95% CI 5.2‐6.4), 4.6 (SD 2.3; 95% CI 4.1‐5.1), and 3.7 (SD 2.0; 95% CI 3.3‐4.1), respectively.

The 95% CIs of the intervention groups did not overlap with those of the control group, indicating that the reductions in anxiety and depression after intervention were stable and precise (Table 7).

**Table 7.** Comparison of Hospital Anxiety and Depression Scale (HADS) anxiety and depression scores among 3 groups of hospitalized patients with lung cancer in a randomized controlled trial of artificial intelligence (AI)–assisted voice cloning education at The First Hospital of Lanzhou University (May 2025 through July 2025), with differences among groups analyzed using 1-way ANOVA.

Time point	Control group (n=58), mean (SD; 95% CI)	Intervention group 1 (n=57), mean (SD; 95% CI)	Intervention group 2 (n=59), mean (SD; 95% CI)	*F* test (*df*)	*P* value
Pre-education anxiety	7.8 (3.2; 7.0-8.6)	7.6 (3.1; 6.8-8.4)	7.7 (3.3; 6.9-8.5)	0.068 (2, 171)	.93
Pre-education depression	6.9 (2.8; 6.2-7.6)	7.1 (2.9; 6.4-7.8)	6.8 (2.7; 6.1-7.5)	0.185 (2, 171)	.83
1 month after discharge anxiety	6.5 (2.9; 5.8-7.2)	5.2 (2.5; 4.6-5.8)	4.3 (2.2; 3.8-4.8)	12.364 (2, 171)	<.001
1 month after discharge depression	5.8 (2.6; 5.2-6.4)	4.6 (2.3; 4.1-5.1)	3.7 (2.0; 3.3-4.1)	13.175 (2, 171)	<.001

## Discussion

### Principal Findings

This RCT investigated the effectiveness of an AI-assisted patient education system integrating voice cloning technology and compared the educational outcomes of physician voice cloning versus patient self-voice cloning. In addition, the study explored the feasibility of using ChatGPT as a supportive tool to assist with evaluating education effectiveness. Consistent with our 3 prespecified hypotheses, the results demonstrated that AI-assisted voice cloning–based education significantly improved education content compliance, knowledge mastery, satisfaction, treatment adherence, and short-term psychological and quality-of-life outcomes compared with traditional education. These findings are consistent with prior research indicating that technology-assisted and personalized education can enhance patient engagement and learning outcomes [10,22]. Notably, education delivered using patients’ own cloned voices yielded superior effects compared with physician voice cloning across multiple outcome domains, supporting the hypothesis that self-referential personalization enhances educational effectiveness.

### Interpretation of Findings and Comparison With Previous Studies

Voice cloning technology represents an emerging application of AI in patient education, offering distinct advantages over conventional education models. By enabling repeated exposure to standardized educational content delivered in a familiar and emotionally resonant voice, voice cloning may enhance attention, comprehension, and memory consolidation [30,31]. Consistent with previous studies demonstrating that familiar auditory cues improve trust and acceptance in health communication, both AI-assisted intervention groups in this study outperformed the traditional education group in education compliance, knowledge mastery, and satisfaction [30,32,33]. Beyond confirming the general effectiveness of voice-based AI education, this study provides novel evidence regarding differences between voice sources. Patients who received education via their own cloned voice achieved higher compliance, satisfaction, knowledge retention, treatment adherence, and better short-term psychological outcomes than those educated using physician voice cloning. Previous studies have largely focused on clinician-narrated or professionally recorded content [34], whereas empirical comparisons involving patient self-voice have been scarce. The observed superiority of self-voice education may be explained by the self-reference effect, whereby information related to the self is processed more deeply and remembered more effectively [17,35,36]. Research in cognitive psychology suggests that self-related stimuli—particularly self-generated or self-similar auditory information—enhance attention, emotional engagement, and memory encoding [16,35]. In this study, hearing one’s own voice narrate medical information may have strengthened self-identification and personal relevance, thereby reinforcing learning and adherence. Furthermore, the self-voice group demonstrated superior outcomes in multiple quality-of-life domains and had greater reductions in anxiety and depression. These findings align with evidence that personalized and self-relevant health communication can enhance perceived control, self-efficacy, and emotional regulation among patients [14,36]. Importantly, the reported effect sizes indicated moderate-to-large intervention effects, suggesting that the observed improvements were not only statistically significant but also clinically meaningful. Reporting both effect sizes and confidence intervals enhances transparency and interpretability and is consistent with CONSORT 2025 recommendations for RCTs [28].

### Implications for AI-Assisted Evaluation of Patient Education

This study also explored the use of ChatGPT as an auxiliary tool to assist with evaluating patient education outcomes based on standardized educational content. Pre-experimental testing demonstrated good agreement between ChatGPT-assisted scoring and expert evaluation, suggesting that LLMs may support structured and reproducible assessment under controlled conditions. These findings are consistent with emerging literature indicating that LLMs can perform semantic analysis and text evaluation tasks with acceptable reliability in medical and educational contexts [6,37]. Compared with traditional manual evaluation, AI-assisted assessment may offer advantages in efficiency, scalability, and consistency, particularly in settings with limited human resources. However, ChatGPT should not be regarded as a fully objective evaluator. Assessment outcomes may still be influenced by prompt design, model architecture, and algorithmic biases. Therefore, AI-assisted evaluation should be considered a complementary tool rather than a replacement for expert assessment, and its use should be accompanied by appropriate human oversight [38,39].

### Study Limitations

Several limitations should be acknowledged. First, the follow-up period was limited to 1 month, restricting conclusions regarding long-term adherence, psychological outcomes, and sustained quality of life benefits. Longer follow-up periods are needed to assess the durability of intervention effects, as recommended in previous digital health education studies [40]. Second, this was a single-center study involving hospitalized patients with relatively homogeneous educational backgrounds, which may limit generalizability. Prior research suggests that educational level and health literacy can influence the effectiveness of digital health interventions [41]. Multicenter studies with more diverse populations are therefore warranted. Third, although voice cloning technology offers a promising approach, current speech synthesis systems still have limitations in emotional nuance and naturalness, which may affect user engagement [13]. Continued technological advancements may further enhance the effectiveness of AI-assisted education. Finally, although AI-assisted education may reduce clinical workload and improve efficiency, this study did not include a formal cost-effectiveness analysis. Future research should systematically evaluate economic outcomes to support large-scale implementation decisions [40]. Because ChatGPT is a continuously updated product, we standardized the scoring workflow (fixed rubric and prompts, prespecified audio format, and single scoring per recording) and provide the complete scoring materials to facilitate reproducibility. Additionally, voice-based delivery systems may misinterpret medication names or specialized terminology, underscoring the need for clinician review of generated content [42].

### Implications for Practice and Policy

Implementation should address unit- and hospital-level factors separately. Targeted strategies should improve staff training, digital access, and policy support. Addressing demographic disparities is key to promoting equity and care quality.

### Conclusions and Broader Implications

This study introduces an innovative patient education model integrating AI voice cloning and ChatGPT, representing a novel approach distinct from previous studies that primarily relied on standard text-to-speech or professionally recorded content. The key innovation lies in using patients’ own cloned voices for health education delivery, leveraging the self-reference effect to enhance learning outcomes. Compared with prior research focusing on clinician-narrated content, this study provides the first empirical evidence that self-voice education produces superior outcomes across multiple domains including compliance, satisfaction, and psychological well-being. These findings contribute to the field by establishing a theoretical and practical framework for personalized AI-driven patient education. In real-world clinical settings, this approach offers a scalable, cost-effective solution to enhance patient engagement, particularly valuable in resource-limited environments where individualized education is challenging to deliver. Future research should focus on multicenter validation, longer follow-up periods, and exploration of optimal voice cloning parameters to maximize educational effectiveness.

## Supplementary material

10.2196/81387 Multimedia Appendix 1 ChatGPT-based compliance scoring: prompts, rubric, and example inputs/outputs.

10.2196/81387 Multimedia Appendix 2 Education content compliance, knowledge mastery, and treatment adherence questionnaires.

10.2196/81387 Multimedia Appendix 3 SF-36 questionnaire.

10.2196/81387 Multimedia Appendix 4 Hospital Anxiety and Depression Scale.

10.2196/81387 Multimedia Appendix 5 Voice cloning patient education prompts, ChatGPT evaluation prompts, example patient knowledge assessment sheet, and notes for researchers.

10.2196/81387 Checklist 1 CONSORT 2025 checklist.

10.2196/81387 Checklist 2 CONSORT-EHEALTH checklist (V 1.6.1).

## Abbreviations

AI: artificial intelligence
CONSORT: Consolidated Standards of Reporting Trials
ITT: intention to treat
LLM: large language model
RCT: randomized controlled trial
