# Supplementary material for: Effectiveness of Al-Assisted Patient Health Education Using Voice Cloning and ChatGPT: Prospective Randomized Controlled Trial
Source: J Med Internet Res. 2026 Mar 19;28:e81387. doi: 10.2196/81387 (PMC13002165; doi:10.2196/81387)
Supplement: Multimedia Appendix 1 [file jmir-v28-e81387-s001.pdf]

ChatGPT-Based Compliance Scoring: Prompts, Rubric, and Example Inputs/Outputs

This appendix provides the complete, prespecified scoring materials used to evaluate the primary outcome (Education Content Compliance Rate) in the randomized controlled trial. The materials are provided to facilitate reproducibility and to comply with CONSORT 2025 and JMIR reporting expectations for AI-assisted methods.

A1. Overview of the Scoring Workflows (Pilot Validation and Main RCT)

Two workflows were evaluated in a separate pilot validation study (January–June 2024) and benchmarked against consensus ratings from three senior nursing experts using the same rubric:

Phase 1 (Verbatim-Matching Workflow): Standard education text + ASR transcript of participant recitation were provided to ChatGPT for rubric-based scoring.
Phase 2 (Semantic Audio-Based Workflow): Standard education text + original audio recording of participant recitation were provided to ChatGPT for rubric-based scoring.
Phase 2 demonstrated higher agreement with expert ratings (weighted κ=.87) than Phase 1 (weighted κ=.72) and was therefore selected for the main RCT.

The main RCT used Phase 2. Each recording was scored once using fixed prompts and the prespecified rubric below; outputs were exported and stored in the trial database.

A2. Input Data Specifications

ASR (Phase 1 only):

Vendor: iFlytek (科大讯飞).
Language/setting: Mandarin in a medical context.
Post-processing: no manual correction of ASR outputs.
Usage: ASR transcript was pasted into ChatGPT as plain text.

Audio (Phase 2; main RCT):

File format: .wav or .mp3.
Sampling rate: 16 kHz (mono recommended).
Typical duration: 60–120 seconds per recording.
Scoring frequency: each audio file was evaluated once (no repeated scoring of the same recording).

A3. Operational Steps in the ChatGPT Web Interface

Phase 1 (pilot only; transcript-based):

1. Open a new ChatGPT web session.
2. Paste the System Prompt (A4) and User Prompt Template (A5).
3. Paste the standard education text into the placeholder [STANDARD TEXT].
4. Paste the ASR transcript into the placeholder [PATIENT RECITATION TEXT].
5. Send once; record the returned total score and item-level scores.

Phase 2 (pilot and main RCT; audio-based):

6. Open a new ChatGPT web session.
7. Paste the System Prompt (A4) and User Prompt Template (A6).
8. Paste the standard education text into the placeholder [STANDARD TEXT].
9. Upload the participant's audio file (WAV/MP3) as an attachment in the same chat.
10. Send once; record the returned total score and item-level scores.

Standardization notes:

The rubric and prompts were fixed across all participants and groups.
The same standard education text corresponding to the participant's clinical pathway was used as reference.
Outputs were recorded without manual adjustment.

A4. Fixed System Prompt (Applied to All Scoring Sessions)

You are an objective clinical rater. Your task is to evaluate how well the participant's recitation covers the key educational content.
Follow the rubric exactly. Do not add medical advice. Do not infer missing information. Score only what is explicitly present.
Return (1) item-level scores, (2) total score, and (3) brief justification per item (≤20 words each).

A5. User Prompt Template (Phase 1: Transcript-Based; Pilot Only)

[STANDARD TEXT]

Participant recitation (ASR transcript; no manual correction):
[PATIENT RECITATION TEXT]

Using the rubric below, score the participant's recitation compared to the standard text.
Rubric:
[RUBRIC]

A6. User Prompt Template (Phase 2: Audio-Based; Pilot + Main RCT)

[STANDARD_TEXT]

Participant recitation is provided as an attached audio file. Listen to the audio and score the participant's recitation compared to the standard text.
Using the rubric below, return item-level scores and total score.
Rubric:
[RUBRIC]

A7. Scoring Rubric (Education Content Compliance Rate)

Each item is scored 0–2. Total score is the sum of item scores. Compliance rate (%) = (Total score / Maximum score) × 100.

Rubric Item	Content Domain	Scoring Anchors
Item 1	Diagnosis/condition overview and purpose of education	0 = absent; 1 = partially covered; 2 = clearly covered
Item 2	Key perioperative or treatment steps	0/1/2 as above
Item 3	Medication or key instructions (if applicable)	0/1/2 as above
Item 4	Warning signs and when to seek help	0/1/2 as above
Item 5	Lifestyle/rehabilitation guidance and follow-up	0/1/2 as above

A8. Example Input/Output (De-identified Illustrative Example)

Example standard education text excerpt (shortened):

"After discharge, take your prescribed medications as directed. Monitor for fever, worsening pain, shortness of breath, or wound redness. Perform breathing exercises daily and attend follow-up in 2 weeks."

Example participant recitation excerpt (illustrative):

"I should take my medicines on time. If I have a fever or breathing gets worse, I need to call the hospital. I will do breathing exercises and come back in two weeks."

Example ChatGPT output format (illustrative):

Item 1: 1/2 – Mentions general instructions, limited condition overview.
Item 2: 1/2 – Refers to discharge steps but not detailed.
Item 3: 2/2 – Clearly states taking medications as directed.
Item 4: 2/2 – Identifies fever and worsening breathing as warning signs.
Item 5: 2/2 – Includes breathing exercises and follow-up timing.
Total: 8/10. Compliance rate: 80%.
