# Supplementary figures and images for: Effectiveness of Al-Assisted Patient Health Education Using Voice Cloning and ChatGPT: Prospective Randomized Controlled Trial
Source: J Med Internet Res. 2026 Mar 19;28:e81387. doi: 10.2196/81387 (PMC13002165; doi:10.2196/81387)

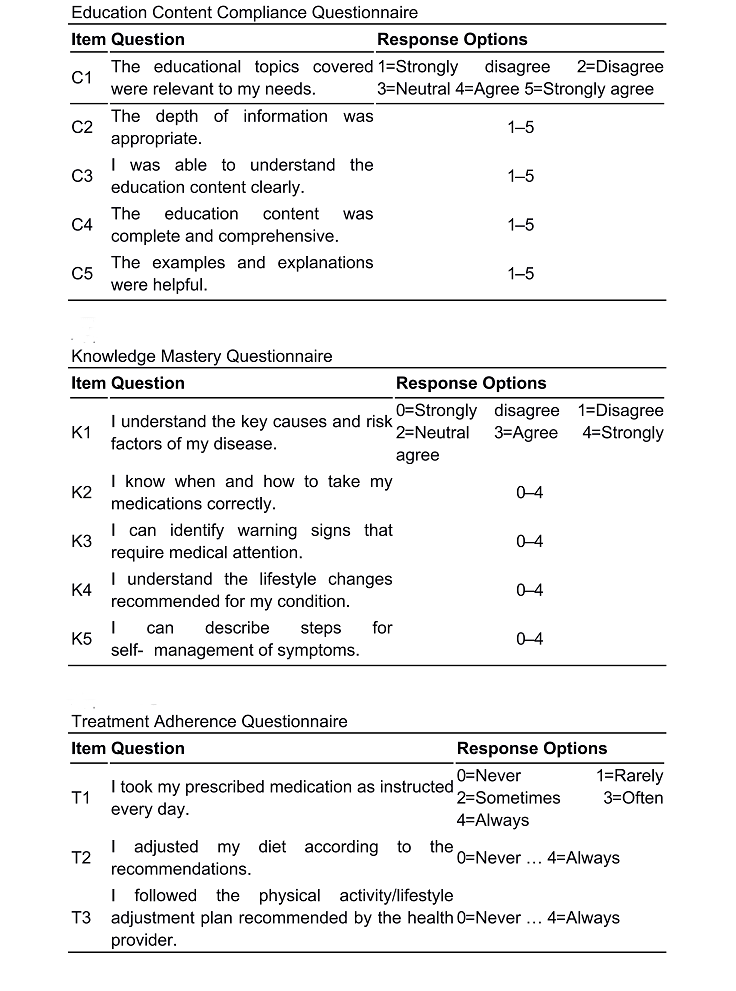

Supplement: Multimedia Appendix 2 [file jmir-v28-e81387-s002.png]

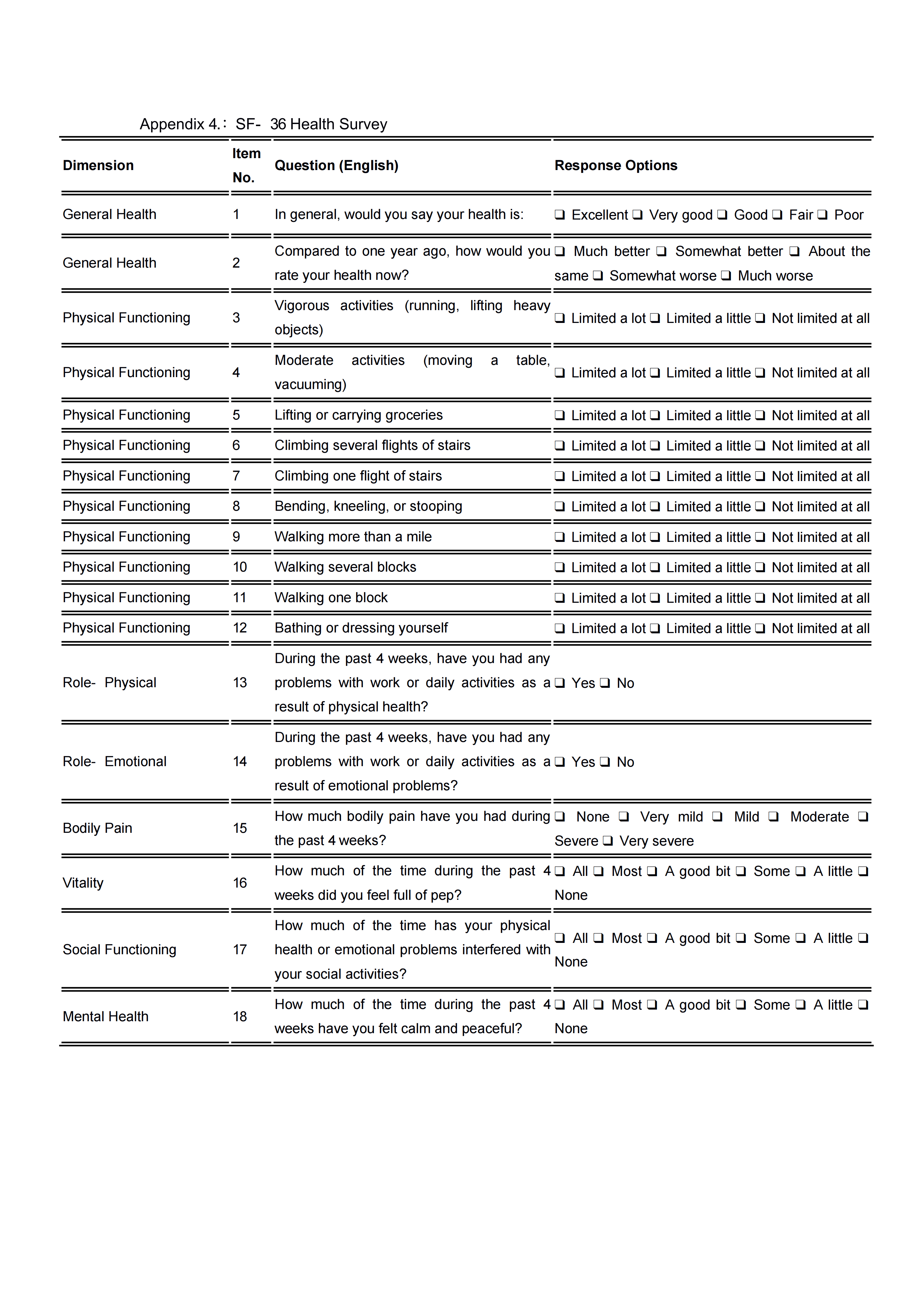

Supplement: Multimedia Appendix 3 [file jmir-v28-e81387-s003.png]

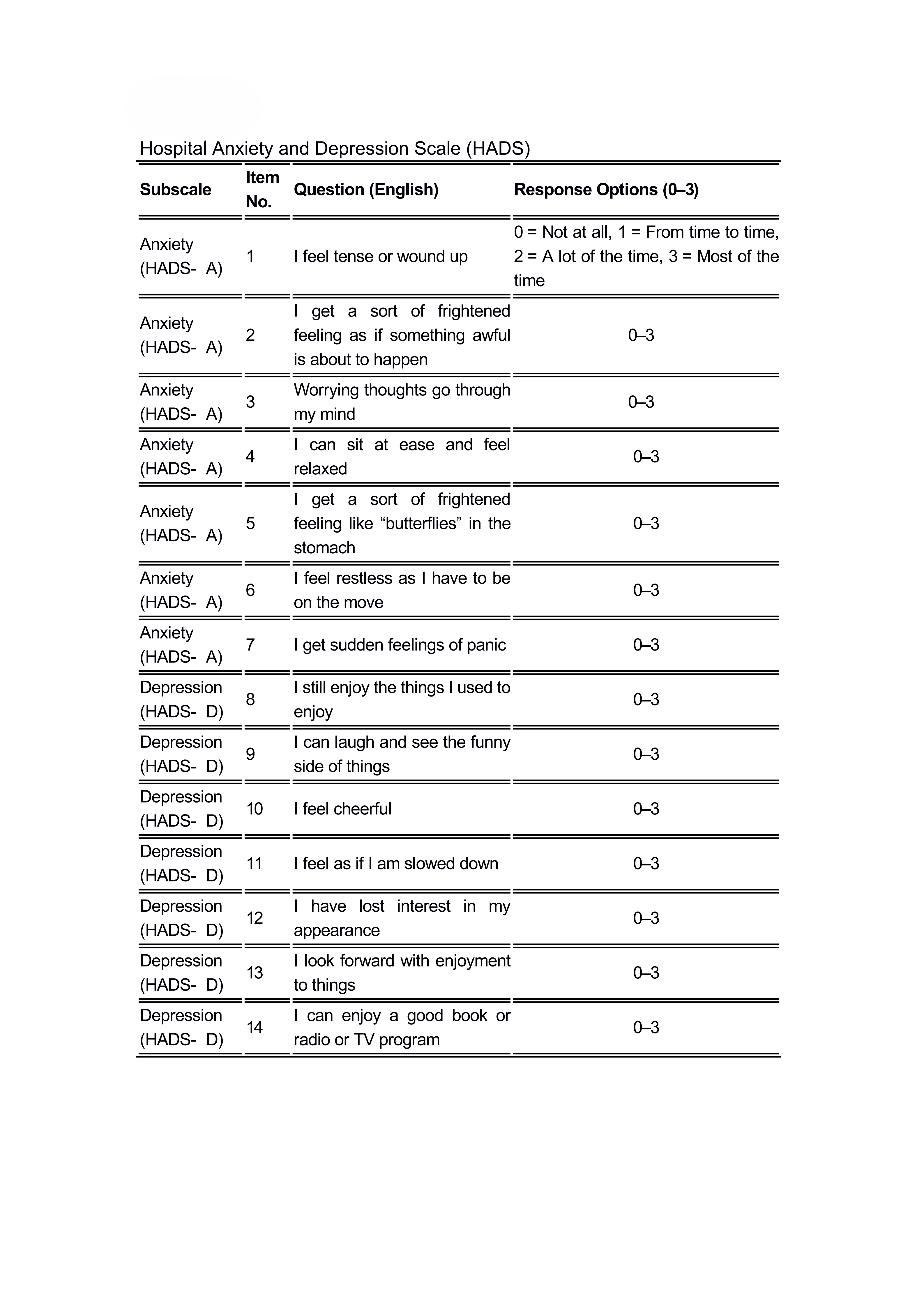

Supplement: Multimedia Appendix 4 [file jmir-v28-e81387-s004.png]

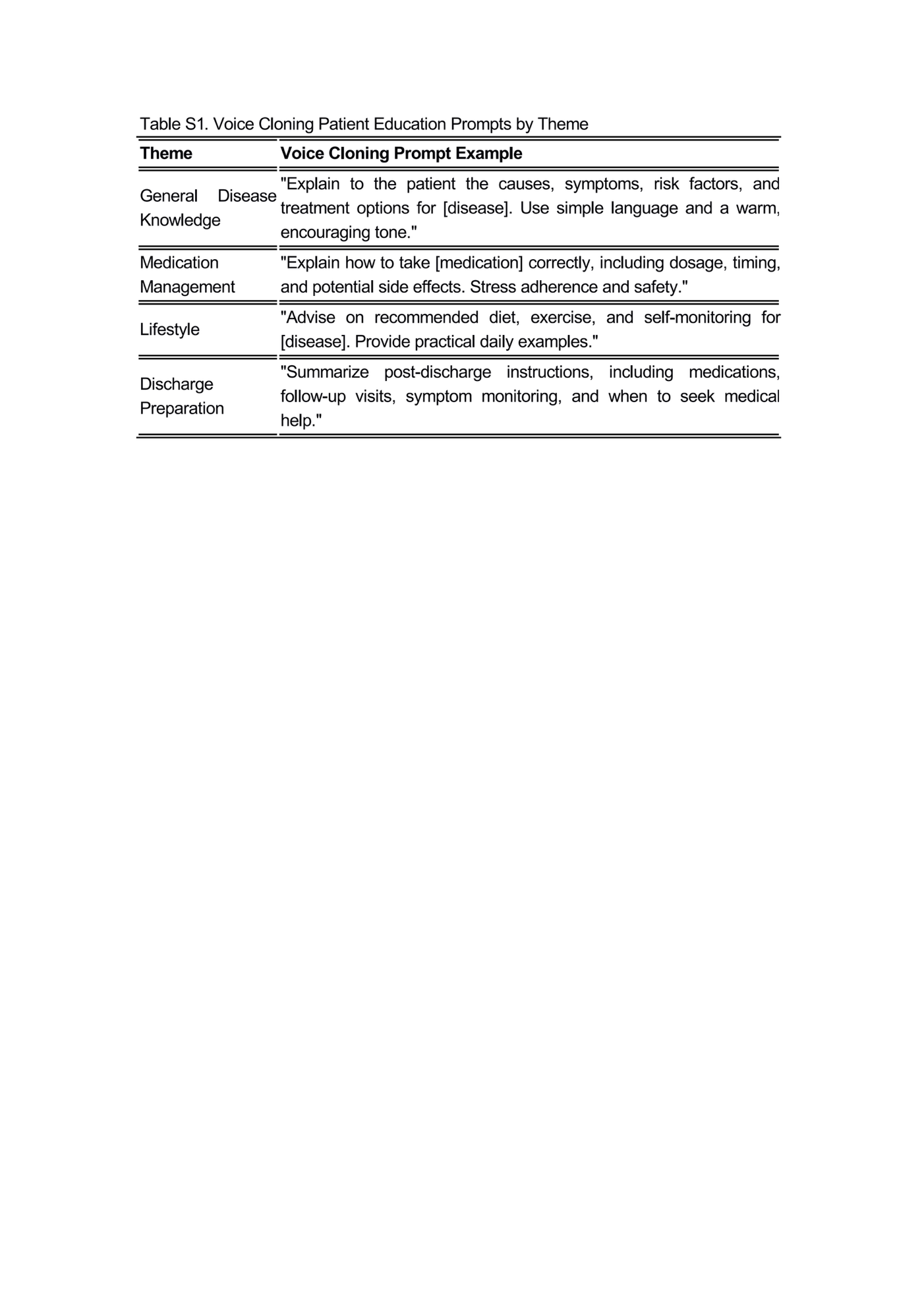


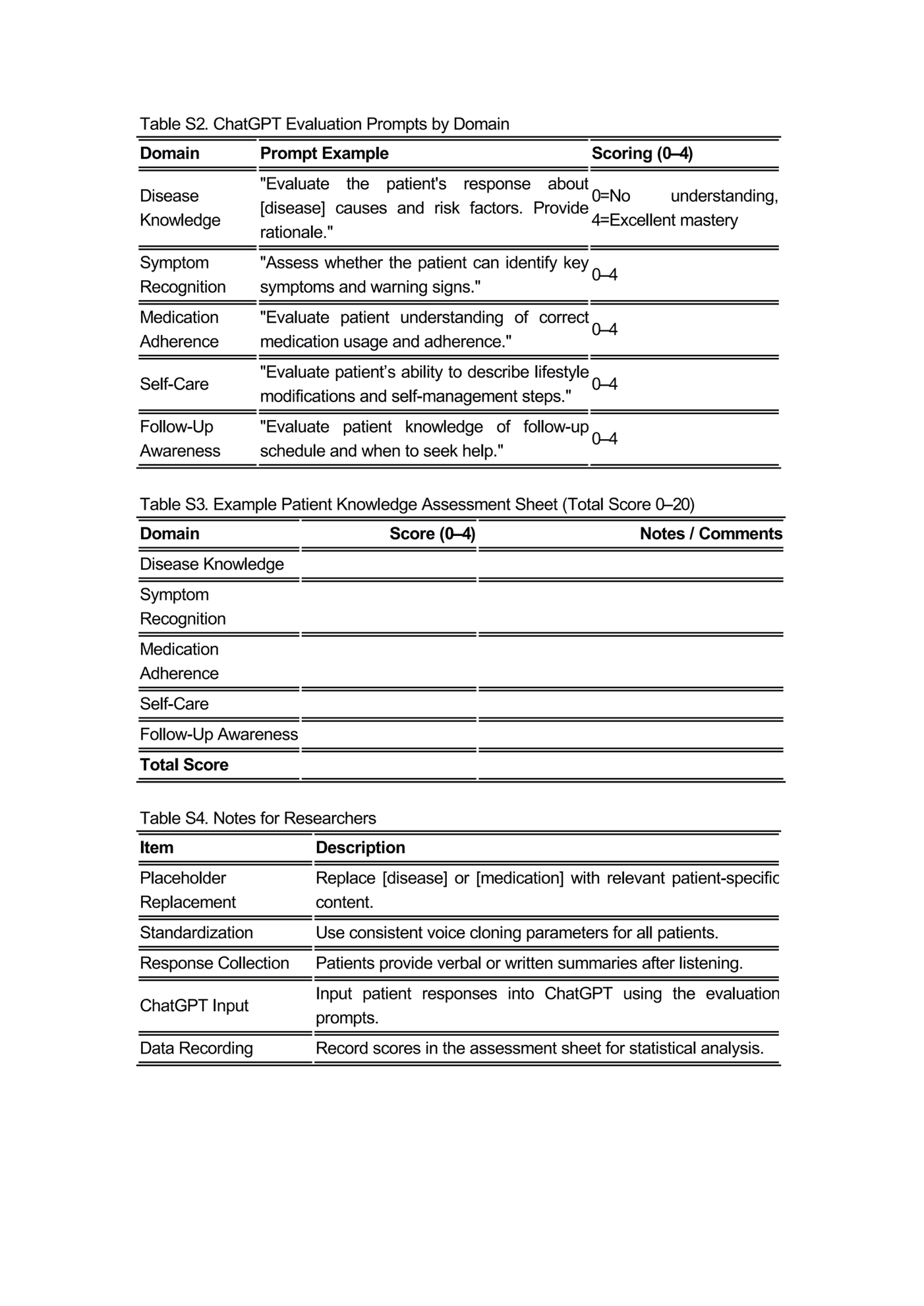

Supplement: Multimedia Appendix 5 [file jmir-v28-e81387-s005.docx]
