# Supplementary material for: Effectiveness of Al-Assisted Patient Health Education Using Voice Cloning and ChatGPT: Prospective Randomized Controlled Trial
Source: J Med Internet Res. 2026 Mar 19;28:e81387. doi: 10.2196/81387 (PMC13002165; doi:10.2196/81387)
Supplement: Checklist 1 [file jmir-v28-e81387-s006.pdf]

CONSORT 2025 Checklist (Completed)

Manuscript Title: A Study on the Effectiveness of AI-Assisted Patient Health Education Using Voice Cloning and ChatGPT: A Prospective Randomized Controlled Trial

Date Completed: January 12, 2026

Journal target: Journal of Medical Internet Research (JMIR)

Section/topic	No	CONSORT 2025 checklist item description	Reported on page no.
**Title and abstract**			
Title and structured abstract	1a	Identification as a randomised trial	Title page (manuscript title)
Title and structured abstract	1b	Structured summary of the trial design, methods, results, and conclusions	Abstract (structured abstract)
**Open science**			
Trial registration	2	Name of trial registry, identifying number (with URL) and date of registration	Abstract (Trial Registration); Methods—Study Design; Methods—Open Science
Protocol and statistical analysis plan	3	Where the trial protocol and statistical analysis plan can be accessed	Methods—Open Science; Methods—Statistical Analysis
Data sharing	4	Where and how the individual de-identified participant data (including data dictionary), statistical code and any other materials can be accessed	Methods—Open Science; Data Availability Statement
Funding and conflicts of interest	5a	Sources of funding and other support (eg, supply of drugs), and role of funders in the design, conduct, analysis and reporting of the trial	Funding
Funding and conflicts of interest	5b	Financial and other conflicts of interest of the manuscript authors	Conflicts of interest
**Introduction**			
Background and rationale	6	Scientific background and rationale	Introduction—Background
Objectives	7	Specific objectives related to benefits and harms	Introduction—Objectives
**Methods**			

Section/topic	No	CONSORT 2025 checklist item description	Reported on page no.
Patient and public involvement	8	Details of patient or public involvement in the design, conduct and reporting of the trial	Abstract (Patient or Public Contribution)
Trial design	9	Description of trial design including type of trial (eg, parallel group, crossover), allocation ratio, and framework (eg, superiority, equivalence, non-inferiority, exploratory)	Methods—Study Design
Changes to trial protocol	10	Important changes to the trial after it commenced including any outcomes or analyses that were not prespecified, with reason	Not applicable (no protocol changes after trial commencement)
Trial setting	11	Settings (eg, community, hospital) and locations (eg, countries, sites) where the trial was conducted	Methods—Participants and Settings
Eligibility criteria	12a	Eligibility criteria for participants	Methods—Participants and Settings
Eligibility criteria	12b	If applicable, eligibility criteria for sites and for individuals delivering the interventions (eg, surgeons, physiotherapists)	Not applicable (single-center trial)
Intervention and comparator	13	Intervention and comparator with sufficient details to allow replication. If relevant, where additional materials describing the intervention and comparator (eg, intervention manual) can be accessed	Methods—Intervention and Procedures; Multimedia Appendix 1
Outcomes	14	Prespecified primary and secondary outcomes, including the specific measurement variable (eg, systolic blood pressure), analysis metric (eg, change from baseline, final value, time to event), method of aggregation (eg, median, proportion), and time point for each outcome	Methods—Outcome Measures; Tables 3–7; Supplementary Table S2

Section/topic	No	CONSORT 2025 checklist item description	Reported on page no.
Harms	15	How harms were defined and assessed (eg, systematically, non-systematically)	Not assessed (educational intervention; no harms anticipated)
Sample size	16a	How sample size was determined, including all assumptions supporting the sample size calculation	Methods—Sample Size
Sample size	16b	Explanation of any interim analyses and stopping guidelines	Not applicable (no interim analyses or stopping guidelines)
Randomisation:			
Sequence generation	17a	Who generated the random allocation sequence and the method used	Methods—Randomization
Sequence generation	17b	Type of randomisation and details of any restriction (eg, stratification, blocking and block size)	Methods—Randomization
			Reported on page no.
Allocation concealment mechanism	18	Mechanism used to implement the random allocation sequence (eg, central computer/telephone; sequentially numbered, opaque, sealed containers), describing any steps to conceal the sequence until interventions were assigned	Methods—Randomization
Implementation	19	Whether the personnel who enrolled and those who assigned participants to the interventions had access to the random allocation sequence	Methods—Randomization
Blinding	20a	Who was blinded after assignment to interventions (eg, participants, care providers, outcome assessors, data analysts)	Methods—Blinding
Blinding	20b	If blinded, how blinding was achieved and description of the similarity of interventions	Methods—Blinding
Statistical methods	21a	Statistical methods used to compare groups for primary and secondary outcomes, including harms	Methods—Statistical Analysis

Section/topic	No	CONSORT 2025 checklist item description	Reported on page no.
Statistical methods	21b	Definition of who is included in each analysis (eg, all randomised participants), and in which group	Methods—Statistical Analysis
Statistical methods	21c	How missing data were handled in the analysis	Methods—Statistical Analysis (Missing data; MICE; Little's MCAR test)
Statistical methods	21d	Methods for any additional analyses (eg, subgroup and sensitivity analyses), distinguishing prespecified from post hoc	Methods—Statistical Analysis
**Results**			
Participant flow, including flow diagram	22a	For each group, the numbers of participants who were randomly assigned, received intended intervention, and were analysed for the primary outcome	Results—Participant Flow; CONSORT Flow Diagram
Participant flow, including flow diagram	22b	For each group, losses and exclusions after randomisation, together with reasons	Results—Participant Flow; CONSORT Flow Diagram
Recruitment	23a	Dates defining the periods of recruitment and follow-up for outcomes of benefits and harms	Abstract (Trial Registration); Methods—Open Science; Results—Recruitment
Recruitment	23b	If relevant, why the trial ended or was stopped	Not applicable (trial completed as planned)
Intervention and comparator delivery	24a	Intervention and comparator as they were actually administered (eg, where appropriate, who delivered the intervention/comparator, how participants adhered, whether they were delivered as intended (fidelity))	Results—Intervention delivery and adherence; Tables 3–7; Supplementary Table S2
Intervention and comparator delivery	24b	Concomitant care received during the trial for each group	Methods—Usual care (control condition)
Baseline data	25	A table showing baseline demographic and clinical characteristics for each group	Results; Table 2 (Baseline characteristics)
Numbers analysed,	26	For each primary and secondary outcome,	Results; Tables 3–7; Supplementary Table

Section/topic	No	CONSORT 2025 checklist item description	Reported on page no.
outcomes and estimation		by group:<br>● the number of participants included in the analysis<br>● the number of participants with available data at the outcome time point<br>● result for each group, and the estimated effect size and its precision (such as 95% confidence interval)<br>● for binary outcomes, presentation of both absolute and relative effect size	S2
Harms	27	All harms or unintended events in each group	Not reported (no harms or adverse events observed/expected)
Ancillary analyses	28	Any other analyses performed, including subgroup and sensitivity analyses, distinguishing pre-specified from post hoc	Results; Supplementary Table S2; Additional analyses (if applicable)
**Discussion**			
Interpretation	29	Interpretation consistent with results, balancing benefits and harms, and considering other relevant evidence	Discussion—Interpretation
Limitations	30	Trial limitations, addressing sources of potential bias, imprecision, generalisability, and, if relevant, multiplicity of analyses	Discussion—Limitations
