# Supplementary material for: Effectiveness of Al-Assisted Patient Health Education Using Voice Cloning and ChatGPT: Prospective Randomized Controlled Trial
Source: J Med Internet Res. 2026 Mar 19;28:e81387. doi: 10.2196/81387 (PMC13002165; doi:10.2196/81387)
Supplement: Checklist 2 [file jmir-v28-e81387-s007.pdf]

CONSORT-EHEALTH Checklist V1.6.1

Manuscript Title: A Study on the Effectiveness of AI-Assisted Patient Health Education Using Voice Cloning and ChatGPT: A Prospective Randomized Controlled Trial

Authors: Yan Sun, Shangqing Xu, Hongying Jin, Xiaoyan Han, Kangqi Jin, Yimei Zhang, Xiaoli Ma, Huaping Wei, Minjie Ma

Trial Registration: Chinese Clinical Trial Registry (ChiCTR2500101882)

Registration Timeline: Application initiated January 15, 2025; finalized April 30, 2025; enrollment began May 2025

Date Completed: January 12, 2026

Item No.	Checklist Item	Reported	Section	Quote/Description
**TITLE AND ABSTRACT**				
1a	Identification as a randomized trial in the title			
1a-i	Identify the mode of delivery in the title	Yes	Title	"AI-Assisted Patient Health Education Using Voice Cloning and ChatGPT"
1a-ii	Mention non-web-based components or co-interventions	Yes	Title	Voice Cloning and ChatGPT as key AI components
1a-iii	Mention primary condition or target group	Yes	Title	"Patient Health Education"
1b	Structured summary of trial			
1b-i	Key features of intervention and comparator in abstract	Yes	Abstract Methods	Three-arm RCT; traditional vs physician voice vs patient self-voice cloning
1b-ii	Level of human involvement in abstract	Yes	Abstract Methods	Traditional education by medical staff vs AI-generated voice with assistant guidance
1b-iii	Open vs closed, web-based vs face-to-face assessments	Yes	Abstract Methods	Tertiary hospital; inclusion/exclusion criteria stated
1b-iv	Results must contain use data	Yes	Abstract Results	174/180 (96.7%); scores with 95% CIs; Cohen's d=0.74; κ=0.87
**INTRODUCTION**				
2a	Scientific background and rationale			
2a-i	Describe the	Yes	Introduction Para 1	Traditional education

	problem and type of system/solution			limitations; AI voice cloning solution
2a-ii	Scientific background, rationale	Yes	Introduction Para 2-3	AI in healthcare; voice cloning; self-reference effect; cites [43,44]
2b	Specific objectives or hypotheses	Yes	Introduction Final Para	Three pre-specified hypotheses clearly stated
**METHODS**				
3a	Trial design including allocation ratio	Yes	Methods, Study Design	Three-arm RCT; 1:1:1 allocation
3b	Important changes to methods	Yes	Methods	No changes; Resemble.ai used consistently
4a	Eligibility criteria			
4a-ii	Open vs closed, recruitment method	Yes	Methods, Participants	Tertiary hospital; May-July 2025
4a-iii	Information given during recruitment	Yes	Methods, Ethical Considerations	Detailed verbal and written information provided
4b	Settings and locations			
4b-i	Report if outcomes self-assessed through questionnaires	Yes	Methods, Data Collection	Trained research assistants collected data
5	Interventions			
5-i	Names, credentials of developers	Yes	Methods, Intervention	Resemble.ai; ChatGPT-4 (OpenAI web interface)
5-ii	History/development process	Yes	Methods, Tool Validation	Independent validation Jan-Jun 2024; 30 volunteers
5-iii	Revisions and updating	Yes	Methods	Phase 1 vs Phase 2; Phase 2 selected for main RCT
5-iv	Quality assurance methods	Yes	Methods, Tool Validation	Phase 1 κ=.72; Phase 2 κ=.87; Cronbach α=.89
5-v	Ensure replicability	Yes	Multimedia Appendix 1	Prompts, rubric, examples provided
5-vii	Access	Yes	Methods, Intervention	Headphones/speakers under research assistant guidance
5-viii	Mode of delivery, features, theoretical framework	Yes	Methods + Introduction	Resemble.ai; ChatGPT; self-reference effect theory
5-ix	Use parameters	Yes	Methods, Intervention	Education time: 20-30 minutes
5-x	Level of human involvement	Yes	Methods, Intervention	Control: verbal by staff; AI: automated with guidance
5-xii	Co-interventions	Yes	Methods, Intervention	Identical content; printed materials provided
6a	Pre-specified outcome measures			
6a-i	Validated instruments	Yes	Methods, Outcome Measures	SF-36, HADS, satisfaction scale, adherence scale

6a-ii	How "use" was defined/measured	Yes	Methods, Outcome Measures	Compliance rate formula provided
6b	Changes to trial outcomes	Yes	Methods	No changes to pre-specified outcomes
7a	Sample size	Yes	Methods, Sample Size	54/group needed; 60/group enrolled (10% dropout)
8a	Sequence generation	Yes	Methods, Randomization	Computer-generated random number table
8b	Type of randomisation	Yes	Methods, Randomization	Simple randomization; 1:1:1 allocation
9	Allocation concealment	Yes	Methods, Randomization	Sealed, opaque envelopes; independent researchers
10	Implementation	Yes	Methods, Randomization	Independent researchers generated sequence and managed envelopes
11a	Blinding			
11a-i	Who was blinded	Yes	Methods, Blinding	Participants/staff not blinded; outcome assessors blinded
11a-ii	Informed consent procedures	Yes	Methods, Ethical Considerations	Written consent; AI voice use disclosed
12a	Statistical methods	Yes	Methods, Statistical Analysis	ITT with MICE (m=5); Little's MCAR test
12b	Additional analyses	Yes	Methods, Statistical Analysis	ANOVA; post hoc tests; sensitivity analyses
X26	Ethics and Informed Consent			
X26-i	Ethics committee approval	Yes	Methods, Ethical Considerations	LDYYLL-2025-805
X26-ii	Informed consent procedures	Yes	Methods, Ethical Considerations	Written consent; data use permission
X26-iii	Safety and security procedures	Yes	Methods, Ethical Considerations	De-identified; encrypted; voice samples deleted
**RESULTS**				
13a	Participant flow (CONSORT diagram)	Yes	Results, Table 1 + Figure 1	200 assessed→180 randomized→174 PP; ITT on 180
13b	Losses and exclusions	Yes	Results + Figure 1	6 lost: transfer(1), consent(3), discharge(1), follow-up(1)
14a	Dates of recruitment and follow-up	Yes	Methods, Study Timeline	May-June 2025 enrollment; July 2025 follow-up
14b	Why trial ended	Yes	Results	Completed as planned
15	Baseline data	Yes	Results, Table 2	Age, gender, education, marital status, disease type, HADS
16	Numbers analysed	Yes	Results + Methods	N=180 ITT; N=174 PP (58+57+59)
17a	Outcomes and estimation	Yes	Results, Tables 3-8	All outcomes with means, SDs, 95% CIs, F-values
17b	Binary outcomes	Yes	Results + Abstract	Cohen's d=0.74; all P-values and 95% CIs

				reported
18	Ancillary analyses	Yes	Results + Suppl Table S2	Pairwise comparisons; ITT results in S2
19	Harms			
19-i	Privacy breaches, technical problems	Yes	Methods, Ethical Considerations	No breaches; voice samples deleted
19-ii	Qualitative feedback	Yes	Results, Table 5	Satisfaction across 5 dimensions
**DISCUSSION**				
20	Limitations	Yes	Discussion, Limitations	Single-center; short follow-up; no participant blinding
21	Generalisability			
21-i	Generalizability to other populations	Yes	Discussion, Limitations	Generalizability limitations discussed
21-ii	Elements different in routine application	Yes	Discussion	Scalability and implementation discussed
22	Interpretation			
22-i	Restate study questions and summarize answers	Yes	Discussion, Principal Findings	Three hypotheses confirmed
22-ii	Unanswered questions, future research	Yes	Discussion, Conclusions	Multi-center trials; longer follow-up suggested
**OTHER INFORMATION**				
23	Registration	Yes	Abstract + Methods	ChiCTR2500101882; Jan 15-Apr 30, 2025; enrollment May 2025
24	Protocol	Yes	Methods	Available upon request
25	Funding	Yes	Manuscript	Funding sources declared
X27	Competing interests	Yes	Conflicts of Interest	No conflicts; no relationship with Resemble.ai/OpenAI

Summary

Section	Total Items	Reported	Not Applicable
Title and Abstract	8	7	1
Introduction	3	3	0
Methods	28	26	2
Results	12	12	0
Discussion	5	5	0
Other Information	4	4	0
**Total**	**60**	**57**	**3**

Completion Rate: 95.0%

Key Manuscript Information

**Trial Registration**	ChiCTR2500101882
**Registration Timeline**	Application: Jan 15, 2025; Finalized: Apr 30, 2025; Enrollment: May 2025
**Ethics Approval**	LDYYLL-2025-805
**Study Period**	May 2025 - July 2025 (enrollment + 1-month follow-up)
**Tool Validation Period**	January - June 2024 (independent study, 30 volunteers)
**Sample Size**	180 randomized; 174 per-protocol; ITT on 180 with MICE
**Allocation Ratio**	1:1:1
**Randomization Method**	Computer-generated random number table
**Allocation Concealment**	Sealed, opaque envelopes; independent researchers
**Blinding**	Outcome assessors/data analysts blinded; participants/staff not blinded
**Primary Outcome**	Education content compliance rate
**Voice Cloning Technology**	Resemble.ai
**AI Evaluation Tool**	ChatGPT-4 (OpenAI web interface)
**Effect Size**	Cohen's d = 0.74
**ChatGPT-Expert Agreement**	Weighted κ = 0.87 (95% CI: 0.82-0.91)
**Missing Data Handling**	ITT with MICE (m=5); Little's MCAR: χ^2=14.5, P=.27

CONSORT Flow Diagram Data (Figure 1)

Stage	Group	n	Details
Assessed for eligibility	-	200	Participants screened
Excluded	-	20	Not meeting criteria (n=8); Declined (n=7); Other (n=5)
Randomized	-	180	Computer-generated sequence (1:1:1)
Allocation: Control	Traditional Education	60	Received intervention as assigned
Allocation: IG1	Physician Voice	60	Received intervention as assigned
Allocation: IG2	Self-Voice	60	Received intervention as assigned
Follow-up: Control	-	58	Lost: Transfer (n=1), Withdrew consent (n=1)
Follow-up: IG1	-	57	Lost: Early discharge (n=1), Withdrew consent (n=2)
Follow-up: IG2	-	59	Lost: Failed follow-

			up (n=1)
Analysis (PP)	-	174	58+57+59; 96.7% completion
Analysis (ITT)	-	180	All randomized with MICE imputation

This checklist was completed based on Manuscript Revised Clean .docx and associated supplementary materials submitted to the Journal of Medical Internet Research.
